# Dynamic equilibrium of skeletal muscle macrophage ontogeny in the diaphragm during homeostasis, injury, and recovery

**DOI:** 10.1038/s41598-024-59527-0

**Published:** 2024-04-21

**Authors:** Qian Li, Feng Liang, Salyan Bhattarai, Maziar Divangahi, Eva Kaufmann, Basil J. Petrof

**Affiliations:** 1 Meakins-Christie Laboratories, Translational Research in Respiratory Diseases Program, Research Institute of the McGill University Health Centre, 1001 Decarie Boulevard, EM3.2224, Montreal, QC H4A 3J1 Canada; 2 Department of Biomedical and Molecular Sciences, Queen’s University, Kingston, ON Canada

**Keywords:** Monocytes and macrophages, Bone marrow transplantation, Inflammation, Acute inflammation, Trauma

## Abstract

The diaphragm is a unique skeletal muscle due to its continuous activation pattern during the act of breathing. The ontogeny of macrophages, pivotal cells for skeletal muscle maintenance and regeneration, is primarily based on two distinct origins: postnatal bone marrow-derived monocytes and prenatal embryonic progenitors. Here we employed chimeric mice to study the dynamics of these two macrophage populations under different conditions. Traditional chimeric mice generated through whole body irradiation showed virtually complete elimination of the original tissue-resident macrophage pool. We then developed a novel method which employs lead shielding to protect the diaphragm tissue niche from irradiation. This allowed us to determine that up to almost half of tissue-resident macrophages in the diaphragm can be maintained independently from bone marrow-derived monocytes under steady-state conditions. These findings were confirmed by long-term (5 months) parabiosis experiments. Acute diaphragm injury shifted the macrophage balance toward an overwhelming predominance of bone marrow (monocyte)-derived macrophages. However, there was a remarkable reversion to the pre-injury ontological landscape after diaphragm muscle recovery. This diaphragm shielding method permits analysis of the dynamics of macrophage origin and corresponding function under different physiological and pathological conditions. It may be especially useful for studying diseases which are characterized by acute or chronic injury of the diaphragm and accompanying inflammation.

## Introduction

Macrophages play several critical roles in the maintenance of skeletal muscle health. This includes the clearance of dead cells, mediation of cytokine responses, secretion of growth factors, and cross-talk with other cell types in the muscle^1,2^. In broad terms tissue-resident macrophages in the adult animal can originate from two primary sources: (1) a macrophage population that is derived postnatally and dependent on continuous replenishment by definitive monocytes released from the bone marrow; and (2) macrophages which are seeded prenatally from embryonic progenitor cells and able to remain independent of the adult bone marrow due to an ability to undergo self-renewal^3–7^. In several tissues examined to date there is evidence that macrophages originating from these two sources can have divergent functional properties under different conditions. Seminal studies over the past decade have also revealed that macrophage ontogeny varies according to organ or tissue type, as well as being altered by aging and disease^3–7^. However, the dynamic balance between these two populations of macrophages and their respective roles in skeletal muscle under different physiological conditions have not been well defined.

Acute skeletal muscle injury leads to a rapid accumulation of macrophages in the tissue, and any interference with the recruitment of adult bone marrow (monocyte)-derived macrophages greatly impedes effective muscle regeneration^8–10^. In addition, several studies using chimeric mice generated through bone marrow transplantation from genetically altered animals have provided key insights into the roles of various genes expressed by bone marrow-dependent macrophages during muscle repair^10–12^. However, an important limitation and confounding factor associated with traditional chimeric mouse models is that whole body irradiation leads to impaired skeletal muscle growth and repair^13,14^. This has been largely attributed to radiation-induced damage to myogenic precursor (satellite) cells and fibro/adipogenic precursors (FAPs), both of which are required for successful muscle regeneration^15–17^. In contrast, there has been little study of the effects of whole body irradiation on resident macrophages in the muscle or whether this intervention alters the balance between bone marrow (monocyte)-dependent and bone marrow-independent macrophages. Furthermore, despite its critical role as the primary muscle of respiration, very little is known about the dynamics of macrophage ontogeny in the diaphragm under different conditions.

Here we report the development of a new approach for generating chimeric mice that protects the diaphragm muscle microenvironment from radiation exposure and thus permits delineation of dynamic changes in macrophage ontogeny. By shielding the local diaphragmatic niche from the adverse effects of irradiation, we were able to assess the relative responses and contributions of bone marrow-dependent versus bone marrow-independent macrophages to the overall macrophage pool under conditions of baseline homeostasis, acute injury, and subsequent recovery.

## Results

### Preservation of satellite cells and resident macrophages by diaphragm shielding

**Figure 1:**
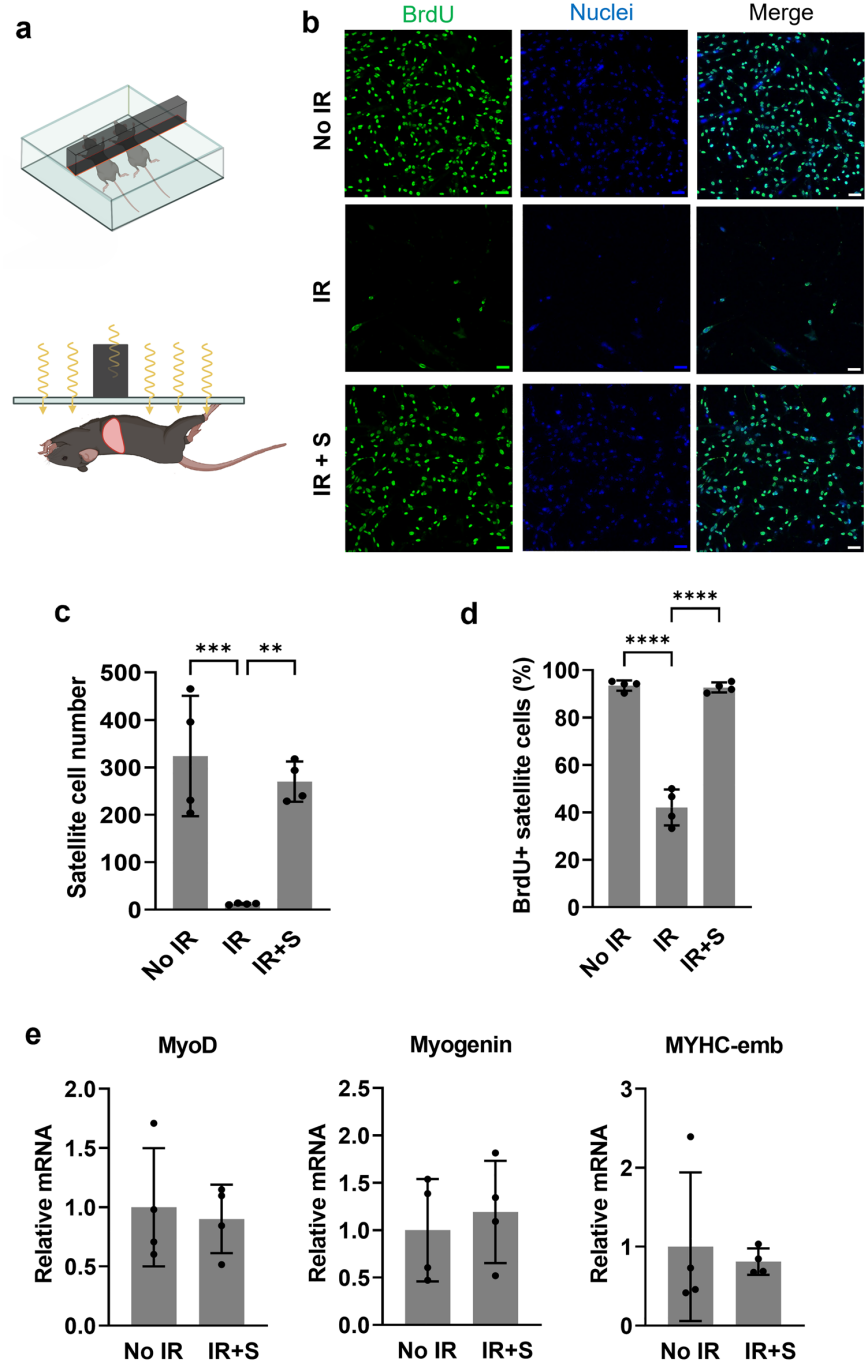
Diaphragm shielding during whole body irradiation preserves satellite cell proliferative capacity. (**a**) Schematic illustration of the shielding procedure used to protect the diaphragm during whole body irradiation. (**b**) Satellite cells were isolated from the diaphragms of mice that were not irradiated (no IR), irradiated without shielding (IR), or irradiated with concurrent diaphragm shielding (IR + S). Representative staining of the cells is shown for BrdU (green) and Hoechst (blue nuclei). Scale bars = 50 μm. (**c**) Quantification of the mean cell number per field of view (n = 4 diaphragms/group, 3 wells/diaphragm, 5–8 photos/well) in diaphragm satellite cell cultures from the 3 groups of mice (**p < 0.01, ***p < 0.001). (**d**) Percentage of BrdU-positive among satellite cells with Hoechst-positive nuclear staining (****p < 0.0001). (**e**) Transcript levels for myogenesis genes in cultured satellite cells obtained from the non-irradiated and shielded groups (n = 4 diaphragms for each group; note that the IR group was not studied due to a lack of satellite cells). All data are expressed as fold-change relative to the non-irradiated (No IR) group mean value.

The experimental set-up employed for protective shielding of the diaphragm during whole body irradiation is depicted in Fig. 1a. Anesthetized mice were placed in a Plexiglas box with their forelimbs raised and a lead bar (2 cm height and 1.5 cm width) positioned over the lower ribcage with 70% of the bar width lying caudal to the xyphoid process. To assess the ability of this shielding to provide radioprotection to diaphragm satellite cells, the latter were isolated from the tissue and placed in culture at 24 h after completing whole body irradiation. After 4 days in high-serum proliferation medium, the number of satellite cells was greatly reduced in the group irradiated without diaphragm shielding in comparison to non-irradiated mice (Fig. 1b,c). In contrast, satellite cell number in the mice irradiated with diaphragm shielding was not significantly different from the non-irradiated control group. In addition, the percentage of diaphragm satellite cells demonstrating BrdU staining did not differ between the non-irradiated and diaphragm-shielded groups, while it was significantly reduced in the unshielded mice (Fig. 1d). We also examined the mRNA expression of several myogenesis genes (MyoD, Myogenin, and MYHC-emb) in the satellite cells cultured in proliferation medium. No differences were observed between the diaphragm-shielded group and non-irradiated controls (Fig. 1e); note that a lack of sufficient satellite cells precluded accurate analysis of gene expression in the mice irradiated without shielding.

**Figure 2:**
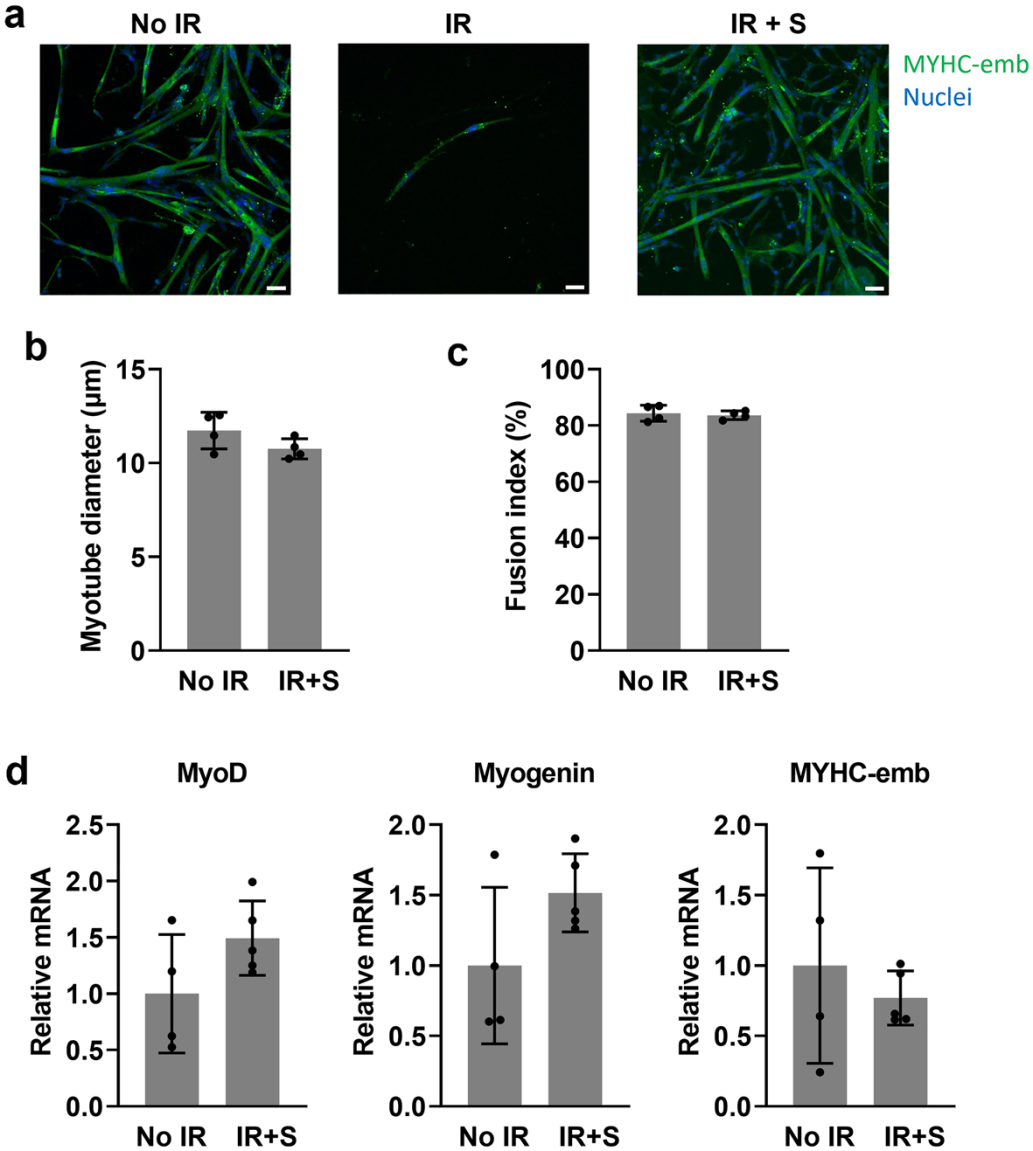
Diaphragm shielding during irradiation preserves satellite cell differentiation capacity. (**a**) Representative images of differentiated myotubes stained for the embryonic isoform of Myosin Heavy Chain (MYHC-emb) and nuclear DNA (Hoechst). Scale bars = 50 μm. (**b**) Quantification of mean myotube diameter (n = 4 diaphragms/group, 3 wells/diaphragm, 3 photos/well). (**c**) Quantification of the myotube fusion index (n = 4 diaphragms/group, 3 wells/diaphragm, 3 photos/well). (**d**) Transcript levels for myogenesis genes in cultured myotubes obtained from the non-irradiated and shielded groups (n = 4 diaphragms/group). All data are expressed as fold-change relative to the non-irradiated (No IR) group mean value.

The capacity of diaphragm satellite cells to differentiate into myotubes was next evaluated by switching the cells to low-serum differentiation medium for 4 days. Myotubes with positive immunostaining for MYHC-emb, a differentiation marker, were widely present in both the non-irradiated and diaphragm-shielded groups, whereas the group irradiated without shielding yielded very few myotubes (Fig. 2a). The average myotube diameter (Fig. 2b) and myotube fusion index (Fig. 2c) did not differ between the non-irradiated and diaphragm-shielded groups, but could not be determined in the group irradiated without shielding due to the very sparse number of myotubes present. The levels of myogenesis gene expression in myotubes derived from the diaphragm satellite cells of non-irradiated and diaphragm-shielded mice did not differ (Fig. 2d).

**Figure 3:**
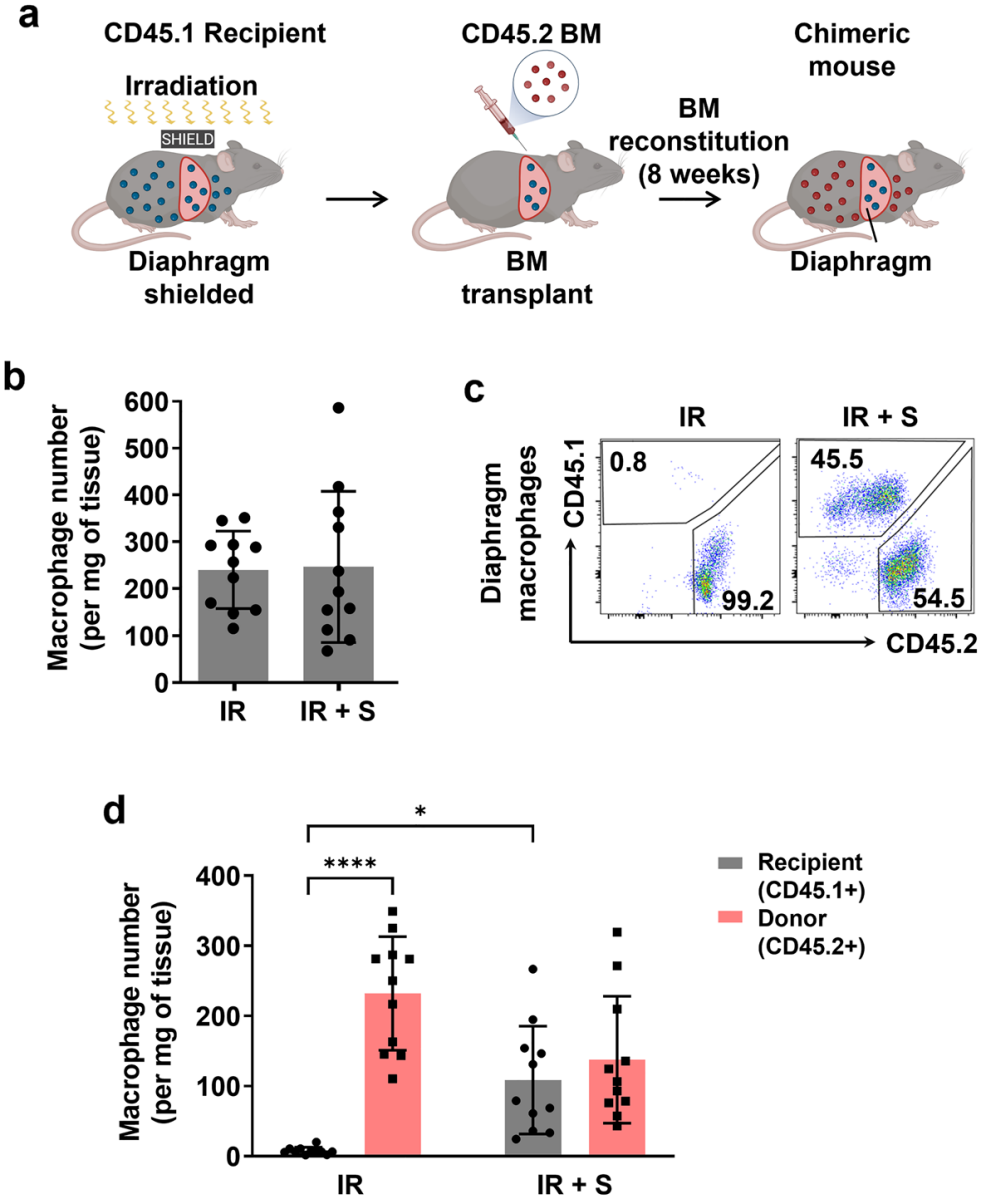
Diaphragm shielding preserves the resident macrophage population after irradiation. (**a**) Chimeric mouse model combined with diaphragm shielding used to study dynamic responses of the bone marrow (monocyte)-derived and local resident macrophage populations in the diaphragm. Irradiated host recipient mice (CD45.1) were transplanted with bone marrow from donor mice (CD45.2) and studied 8 weeks later. (**b**) Absolute number of diaphragm macrophages in the unshielded (IR) and shielded (IR + S) groups at 8 weeks after bone marrow transplantation (n = 11/group). (**c**) Representative examples of flow cytometry plots which identify macrophages of either bone marrow donor (CD45.2) or host recipient (CD45.1) origin in diaphragms that were either unshielded (IR) or shielded (IR + S) during irradiation. Macrophages were defined as CD45+ (either CD45.1 or CD45.2), SiglecF-negative, CD11c-negative, CD11b+, and F480+. (**d**) Absolute quantification of bone marrow donor versus host recipient origin macrophages in diaphragms that were either unshielded (IR) or shielded (IR + S) during irradiation (n = 11/group; *P < 0.05, ****p < 0.0001).

To determine the impact of whole body irradiation on resident macrophages in the diaphragm and the ability of our shielding procedure to protect these cells, we next generated chimeric mice by transplanting bone marrow from CD45.2 allele donor mice into irradiated CD45.1 allele host recipient mice as illustrated in Fig. 3a. The mice were studied at 8 weeks after the procedure to allow sufficient time for bone marrow reconstitution. Although the absolute number of macrophages in the diaphragm (normalized to muscle weight, which was ~ 20% lower in the unshielded group) did not differ between the unshielded and shielded groups (Fig. 3b), flow cytometry revealed dramatic differences in their ontological composition. In the group irradiated without shielding, the original host recipient (CD45.1) macrophages in the diaphragm were almost completely eliminated and replaced by donor origin (CD45.2) macrophages from the transplanted bone marrow (Fig. 3c,d). In contrast, for the shielded group approximately half of the macrophages in the diaphragm retained their initial CD45.1 host recipient origin. Taken together, the above findings indicate that diaphragm shielding promotes a more physiologically relevant microenvironment in the muscle by preserving satellite cell functionality and preventing elimination of the original tissue-resident macrophage population.

### A major population of resident macrophages in the diaphragm does not rely on bone marrow-derived monocytes for replenishment

**Figure 4:**
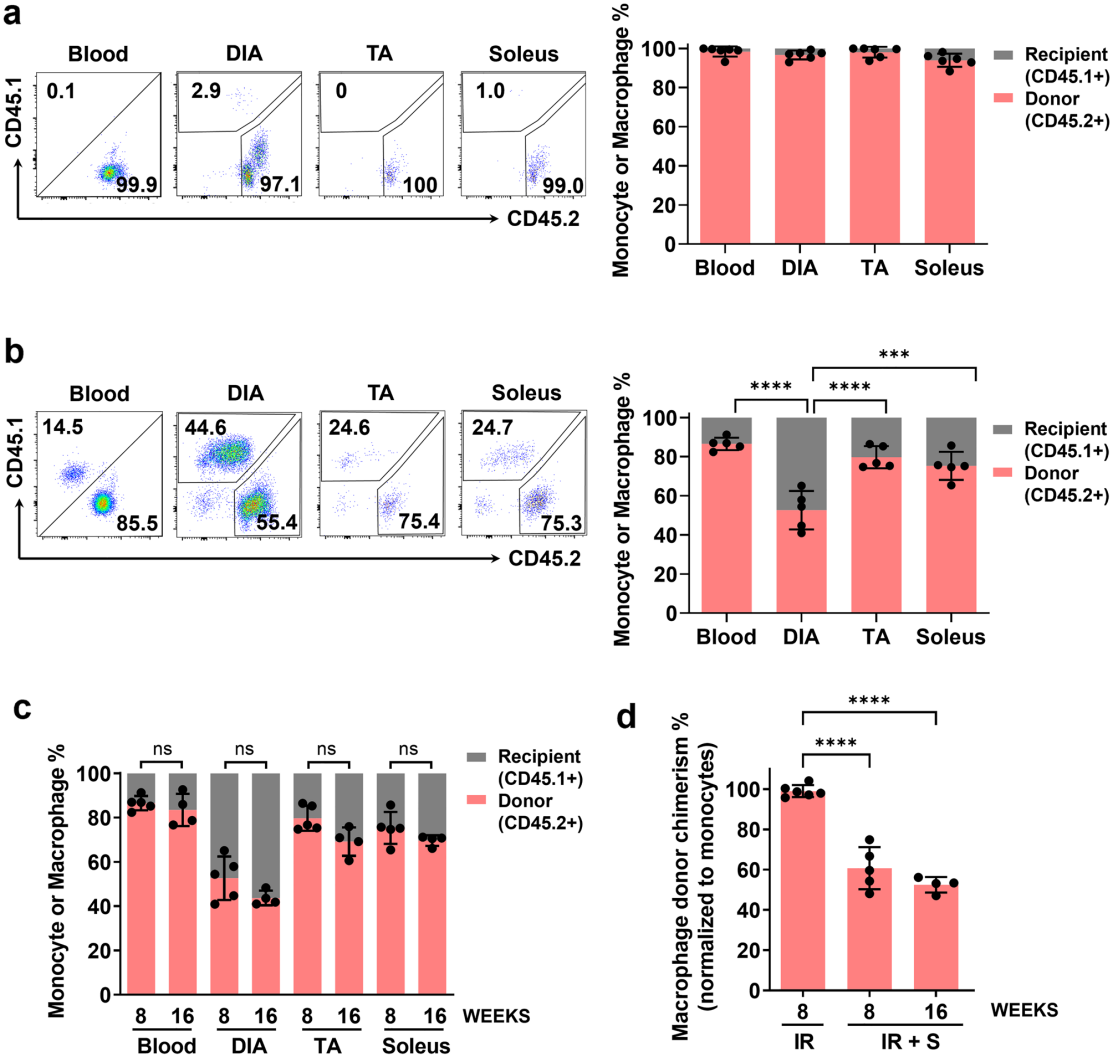
Diaphragm shielding reveals the existence of a major bone marrow (monocyte)-independent macrophage population. (**a**) Representative flow cytometry plots (left) and group mean data (right) show the relative percentages of CD45.2+ (donor) versus CD45.1+ (recipient) monocytes (Blood) and macrophages in the diaphragm (DIA), tibialis anterior (TA), and soleus muscles at 8 weeks after bone marrow transplantation without diaphragm shielding (IR) (n = 6 mice/group). (**b**) Same analysis as in (**a**) performed with diaphragm shielding (IR + S) (n = 5 mice/group; ***P < 0.001, ****P < 0.0001). (**c**) In diaphragm-shielded mice, the relative percentages of CD45.2+ versus CD45.1+ monocytes (Blood) or macrophages (DIA, TA, and soleus muscles) are shown at 8 weeks and 16 weeks post-transplantation (n = 4–5 mice/group; ns = not statistically significant). (**d**) Normalized diaphragm macrophage chimerism, determined as the percentage of donor (CD45.2+) macrophages in the muscle divided by the percentage of CD45.2+ blood monocytes in each animal. The analyses were performed in unshielded mice (IR) and diaphragm-shielded mice (IR + S) at either 8 or 16 weeks after bone marrow transplantation (n = 4–6 mice/group; ****P < 0.0001).

We next sought to explore the relationship between monocyte-derived macrophages and the global tissue-resident macrophage pool which exists in the diaphragm at steady-state. In CD45.1 host recipients transplanted with bone marrow from CD45.2 donors, we directly compared the resulting chimerism levels for monocytes in the blood and macrophages in the muscle. This analysis was performed for the diaphragm and two hindlimb muscles, the fast-twitch tibialis anterior (TA) and the slow-twitch soleus (note that these hindlimb muscles were fully exposed to irradiation in diaphragm-shielded as well as unshielded mice). If all macrophages in the muscle tissue are derived from blood monocytes, one would expect the steady-state chimerism levels (relative percentages of CD45.2 versus CD45.1) in blood monocytes and muscle macrophages to be very similar. This was the case for unshielded mice, where the percentage of host-origin monocytes in the blood was very low (~ 2%) and closely matched the percentage of host-origin macrophages in the diaphragm (~ 3%) at 8 weeks after bone marrow transplantation (Fig. 4a). Similarly the hindlimb muscles, which were fully exposed to irradiation in all mice (both with and without diaphragm shielding), showed closely matching chimerism levels for blood monocytes and muscle macrophages.

On the other hand, the diaphragms of shielded mice at 8 weeks after bone marrow transplantation exhibited a disproportionately high percentage of host-origin macrophages compared to the corresponding host-origin blood monocytes (Fig. 4b). The latter showed a higher background percentage than in unshielded mice due to the partial radioprotection of underlying thoracic bones by the shielding. Nevertheless, the highly significant difference between diaphragm and blood host-origin levels (~ 47% versus ~ 13%, respectively; P < 0.0001) in the shielded mice implicates monocyte-independent macrophages as a major contributor to the normal tissue-resident macrophage population of the diaphragm.

To investigate the stability of macrophage ontogeny over time, we also followed the levels of monocyte and macrophage chimerism in diaphragm-shielded mice out to 16 weeks after bone marrow transplantation. There were no significant differences in the levels of monocyte or macrophage chimerism between 8 and 16 weeks post-transplantation (Fig. 4c). These findings imply that under normal homeostatic conditions and over an extended period of time (i.e., for at least 4 months post-transplantation), a significant component of the resident macrophage pool in the shielded diaphragm was able to remain stably independent from bone marrow monocytes as a source of replenishment.

Macrophage chimerism in the muscle was also normalized to the corresponding level of monocyte chimerism to allow comparisons of the predicted monocyte contribution to tissue-resident diaphragm macrophages across the different conditions (Fig. 4d). This analysis indicated that when the diaphragm is irradiated without protective shielding, there is a near total dependence (~ 99%) on blood monocytes for subsequent muscle macrophage replacement. In contrast, diaphragm shielding reduced this predicted monocyte-dependence to the range of 50–60%, suggesting that the remainder of the macrophages are potentially monocyte-independent.

### Parabiosis confirms long-term maintenance of a major bone marrow (monocyte)-independent macrophage population in skeletal muscles at steady-state

**Figure 5:**
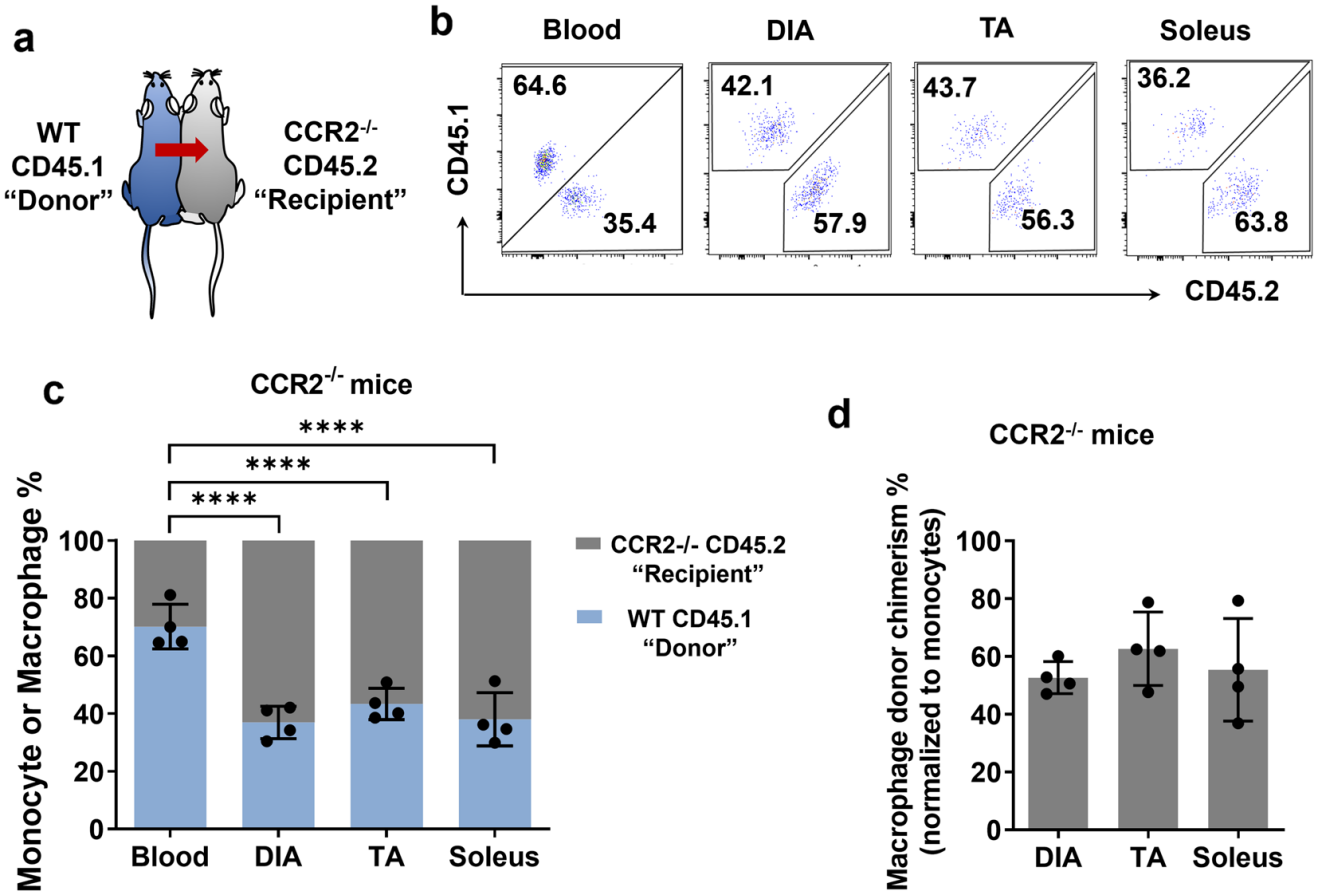
Parabiosis indicates bone marrow (monocyte)-independent macrophages are stably maintained in the diaphragm and other skeletal muscles. (**a**) Illustration of parabiosis model. The wild-type (WT) CD45.1 mouse acts as a de facto monocyte donor (represented by the arrow) to the recipient CCR2−/− CD45.2 mouse. (**b**) Representative FACS plots after 5 months of parabiosis showing the relative percentages of CD45.1+ (donor) and CD45.2+ (recipient) monocytes or macrophages in the blood, diaphragm (DIA), tibialis anterior (TA), and soleus muscles of CCR2−/− mice. (**c**) Group mean data are shown for the relative percentages of CD45.1+ and CD45.2+ monocytes or macrophages in the blood, diaphragm (DIA), tibialis anterior (TA), and soleus muscles in CCR2−/− mice after 5 months of parabiosis (n = 4 parabiotic pairings; ****P < 0.0001). (**d**) Normalized muscle macrophage chimerism levels in CCR2−/− mice, determined as the percentage of donor (CD45.1+) muscle macrophages divided by the percentage of CD45.1+ blood monocytes in each animal (****P < 0.0001).

To validate our findings in the diaphragm-shielded chimeric mice, we next conducted parabiosis experiments in which the blood circulations of CD45.1 allele wild-type (WT) mice and CD45.2 allele CCR2-deficient (CCR2−/−) mice were surgically joined. Since CCR2−/− mice have impaired monocyte release from the bone marrow^18,19^, the WT mouse effectively acts as a monocyte “donor” to the paired “recipient” CCR2−/− mouse (Fig. 5a). Accordingly, after 5 months of parabiosis the percentage of WT donor-origin (CD45.1) monocytes was greater than the recipient-origin (CD45.2) monocytes in the blood circulation of CCR2−/− mice (~ 69% versus ~ 31%, respectively; P < 0.0001). However, in keeping with the data in diaphragm-shielded chimeric mice, the diaphragm macrophage population of CCR2−/− parabionts contained a disproportionately high percentage of recipient-origin macrophages relative to the corresponding blood monocyte levels (Fig. 5b,c). Similar findings were obtained for the limb muscles (TA and soleus) of CCR2−/− parabionts. Normalization of the muscle macrophage chimerism levels to their corresponding blood monocyte chimerism level predicted that only ~ 50–60% of the steady-state macrophage pool in the diaphragm (as well as limb muscles) was monocyte-dependent (Fig. 5d). Therefore, the findings obtained in diaphragm-shielded chimeric mice and in the parabiosis model were consistent with one another, both suggesting that up to almost half of the macrophages in the diaphragm are monocyte-independent under normal steady-state conditions.

### Bone marrow-derived macrophages are responsible for the increase in diaphragm macrophages after acute injury

**Figure 6:**
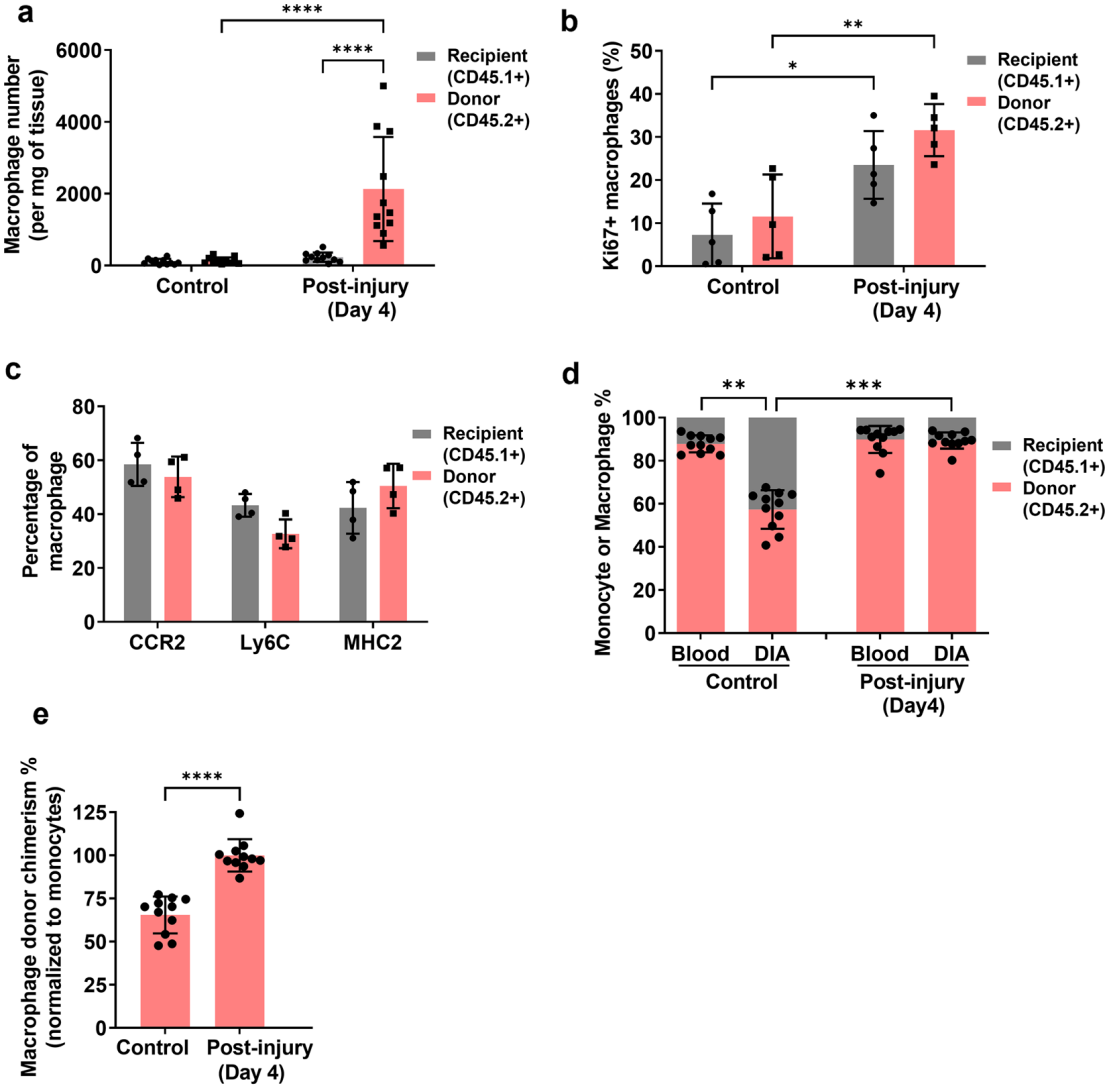
Comparison of bone marrow-dependent versus bone marrow-independent macrophages after acute injury in the shielded diaphragm. (**a**) Absolute cell number (per mg of tissue) for diaphragm macrophages of donor (CD45.2) or host recipient (CD45.1) origin for uninjured control mice versus cardiotoxin-treated mice in the shielded diaphragm at day 4 post-injury (n = 11 mice/group; **** P < 0.0001). (**b**) Ki67 expression determined by flow cytometry in diaphragm macrophages of donor or host recipient origin under the same experimental conditions described in (**a**) (n = 5 mice/group; ****P < 0.0001). (**c**) Phenotypic marker expression (CCR2, Ly6C, MHC2) determined by flow cytometry in diaphragm macrophages of donor or host recipient origin under the same experimental conditions described in (**a**) (n = 4 mice/group). (**d**) Relative percentages of donor (CD45.2) or host recipient (CD45.1) monocytes (Blood) and macrophages in the shielded diaphragm (DIA) (n = 11 mice/group; **P < 0.01, ***P < 0.001). (**e**) Diaphragm macrophage donor chimerism level (normalized to the percentage of donor blood monocytes) for the same mice depicted in (**c**) (****P < 0.0001).

We next employed diaphragm-shielded chimeric mice (8 weeks after bone marrow transplantation) to determine the dynamics of the two macrophage populations after acute injury. Although acute necrotic injury of skeletal muscle is known to cause a massive elevation in the number of intramuscular macrophages, the precise contribution of bone marrow-dependent versus bone marrow-independent macrophages is unclear. Diaphragm injury was achieved by exposing the muscle via laparotomy to the necrosis-inducing agent cardiotoxin, followed by muscle harvest 4 days later. The absolute number of bone marrow donor-origin (CD45.2) macrophages per mg of diaphragm tissue increased by approximately 15-fold post-injury, whereas the host-origin (CD45.1) macrophage number remained unchanged (Fig. 6a).

To evaluate whether augmented cellular proliferation could contribute to the increased numbers of diaphragm macrophages following injury, Ki67 expression was assessed using flow cytometry (Fig. 6b). In uninjured diaphragms, the baseline percentage of Ki67-positive cells was similar in the CD45.1 and CD45.2 macrophage populations. Although both CD45.1 and CD45.2 macrophages demonstrated an increased frequency of Ki67-positive cells following acute diaphragm injury, the levels did not differ significantly between the two macrophage populations. In addition, the percentages of cells expressing the phenotypic markers CCR2, Ly6C, and MHC (major histocompatibility complex) 2 were also not different between the CD45.1 and CD45.2 macrophage populations at day 4 post-injury (Fig. 6c).

Comparisons were made between the percentages of host-origin macrophages in the shielded diaphragm and the corresponding host-origin blood monocytes (Fig. 6d). The normalized macrophage chimerism levels in the muscle once again predicted that ~ 60% of macrophages in the uninjured shielded diaphragm were of bone marrow (monocyte)-derived origin, whereas this value rose to nearly 100% in the cardiotoxin-injured group (Fig. 6e). Taken together, these findings indicate that increased monocyte recruitment from the bone marrow, rather than increased local proliferation of monocyte-independent macrophages, is overwhelmingly the main source of increased macrophage numbers in the early period following acute necrotic injury of the muscle.

### The pre-injury pattern of macrophage ontogeny is restored in healed diaphragm muscle

**Figure 7:**
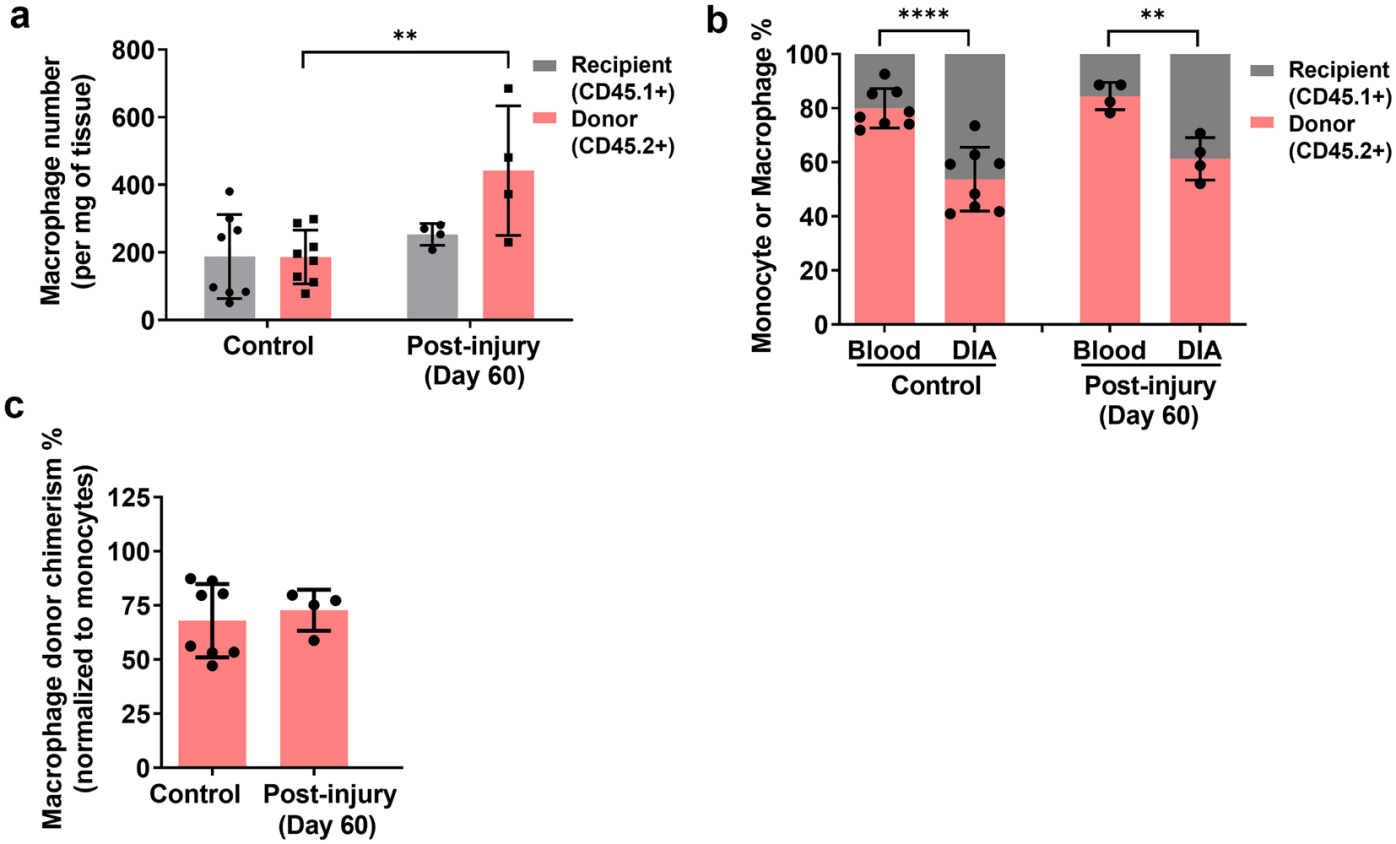
Comparison of bone marrow-dependent versus bone marrow-independent macrophages following recovery from diaphragm injury. (**a**) Absolute cell number (per mg of tissue) for diaphragm macrophages of donor (CD45.2) or host recipient (CD45.1) origin for uninjured control mice versus cardiotoxin-treated mice in the shielded diaphragm at day 60 post-injury (n = 8 and 4 mice/group, respectively; **P < 0.01, ****P < 0.0001). (**b**) Relative percentages of donor (CD45.2) or host recipient (CD45.1) monocytes (Blood) and macrophages in the diaphragm (DIA) under the same experimental conditions described in (**a**). (**P < 0.01, ****P < 0.0001). (**c**) Diaphragm macrophage donor chimerism (normalized to the percentage of donor blood monocytes) is shown for uninjured control mice versus cardiotoxin-treated mice at 60 days post-injury.

To ascertain whether the ontological composition of macrophages in the diaphragm returns to normal once the muscle has recovered from acute injury, we analyzed diaphragm-shielded chimeric mice which had undergone cardiotoxin treatment 60 days earlier. The absolute number of bone marrow-origin macrophages was mildly increased at 60 days post-injury compared to age-matched uninjured mice (Fig. 7a). However, the monocyte-normalized macrophage chimerism levels in the diaphragm did not differ between these two groups (Fig. 7b,c). Therefore, these data suggest that once the diaphragm has undergone complete regeneration and recovery from acute necrotic injury, the normal pre-injury proportionality between bone marrow-dependent and bone marrow-independent macrophages is largely restored within the new tissue-resident population.

## Discussion

The diaphragm is a unique skeletal muscle not only in terms of its continuous (24 h per day) rhythmic activation during the act of breathing, but also with respect to its anatomical barrier function between the thoracic and abdominal cavities^20,21^. Acute or chronic damage to the diaphragm occurs under different pathological conditions including pulmonary disorders^22,23^, sepsis^24^, mechanical ventilation^25^, and in primary muscle diseases such as muscular dystrophies^26–28^. Despite the critical importance of the diaphragm for survival, there has been relatively little study of diaphragm muscle regeneration and to our knowledge no previous evaluation of macrophage ontogeny during diaphragm injury and repair.

Before discussing our results in detail, certain limitations of this study should be acknowledged. A key feature of our methodology was the use of lead shielding to protect the diaphragm from pre-transplantation irradiation and thus maintain a physiologically intact muscle tissue microenvironment. A disadvantage of diaphragm shielding is that portions of the rib cage and spine are also protected from radiation exposure, so that the recipient host bone marrow is not entirely ablated. As a result the donor bone marrow contribution to blood monocytes in our study was less than 100% (i.e., in the range of 85–90%). We corrected for this factor by normalizing the percentage of donor macrophages in the muscle to the corresponding percentage of donor monocytes in the blood as previously described^29,30^. Nevertheless, we cannot exclude the possibility that this minor residual population of bone marrow-dependent macrophages from the host was somehow able to influence the behavior of the larger host resident macrophage population that is bone marrow-independent. In addition, the time required for full equilibration between the blood and muscle tissue compartments is unknown and could vary depending on factors such as macrophage proliferation, lifespan and the exchange rate with monocytes. However the fact that very similar results were obtained with the diaphragm shielding and parabiosis models suggests that by 8 weeks, full equilibration was achieved. While the basic premise of our model is that lead shielding effectively protects the local diaphragm tissue niche from harmful radiation effects, we recognize that systemic inflammation associated with whole body irradiation could still theoretically impact the function of various cell types within the shielded muscle^31^.

Prior investigations have made extensive use of whole body irradiation followed by bone marrow transplantation to study the roles of different genes in monocyte-derived macrophages during muscle regeneration^10–12^. However, radiation exposure has major adverse effects on satellite cell function and hence muscle regeneration, thus introducing an important confounding factor in such studies^13,14^. Diaphragm shielding, in contrast, maintains the proliferation and differentiation capacities of satellite cells at the same levels observed in the non-irradiated diaphragm as reflected by cell number, BrdU incorporation, myotube size, fusion index and the expression of several prototypical genes involved in myogenesis. Furthermore, while satellite cell dysfunction due to irradiation is a known phenomenon, the impact of radiation exposure on skeletal muscle resident macrophages has not been equally recognized. Although certain resident macrophage populations in other tissues are relatively radio-resistant^6^, in the absence of protective shielding almost no tissue-resident macrophages of host origin could be found in the diaphragm at 8 weeks post-irradiation. Therefore, any radio-resistant macrophage population that might exist in skeletal muscle appears to be exceedingly small. Accordingly, our findings predict that in patients subjected to whole body irradiation^13^, the normal homeostatic balance would be disrupted to overwhelmingly favour monocyte-dependent macrophages within the newly established post-irradiation resident macrophage population.

In healthy non-irradiated mice, the ontological composition of macrophages under normal steady-state conditions is known to vary across different tissues and organs^3^. For instance, microglia in the brain are derived from the embryonic yolk sac and self-renew in the adult with minimal input from blood monocytes. On the other hand, intestinal lamina propria macrophages are mostly dependent on blood monocytes for their replenishment. To date there has been little examination of macrophage ontogeny in skeletal muscle compared to other tissues. A recent fate-mapping study reported that about 60% of resident macrophages are monocyte-derived in mouse skeletal muscles at 26 weeks of age^32^. This is consistent with the results we obtained with diaphragm shielding, where the blood monocyte-normalized muscle macrophage chimerism levels also predicted that ~ 60% of the resident macrophage population is bone marrow (monocyte)-dependent at steady-state. These findings were further corroborated by our long-term (5 months) experiments in the parabiosis model, which to our knowledge represent the first use of parabiosis to elucidate macrophage ontogeny in skeletal muscle.

Following acute cardiotoxin injury, the number of bone marrow (monocyte)-derived macrophages increased massively to represent almost 100% of diaphragm macrophages, whereas the number of bone marrow-independent macrophages was not significantly altered. This striking difference is primarily attributable to increased recruitment rather than a greater proliferation rate of the bone marrow-derived macrophages, since Ki67-positivity did not differ between the two macrophage populations. In addition, the large dichotomy in numerical contributions of bone marrow-dependent and bone marrow-independent macrophages suggests that these cells have different functional roles in the early skeletal muscle repair process.

Another fundamental and previously unexamined issue is the question of what happens to the steady-state levels of macrophages of distinct origins following recovery from skeletal muscle injury. For instance, monocyte-derived macrophages have been found to significantly replace the original cardiac tissue resident macrophage population that is depleted following acute myocardial infarction^29,33^. A similar phenomenon has been described following bleomycin-induced lung injury^34^. In the liver, the long-term persistence of monocyte-derived macrophages and their ability to be converted into resident Kupffer cells appears to depend on the nature of the injury^35,36^. Here we sought to determine whether the initial cardiotoxin injury-induced influx of monocyte-derived macrophages leads to a sustained increase in the proportion of these cells in the diaphragm after recovery from injury, which would fundamentally alter the ontogeny of the reconstituted resident macrophage pool. Our findings do not support this scenario. At 60 days post-injury when the inflammatory response has resolved and skeletal muscle regeneration is considered complete, we found that the relative proportions of bone marrow-dependent versus bone marrow-independent macrophages had largely returned to the control levels of uninjured muscle.

The precise mechanisms regulating these responses are poorly understood, but may be influenced in part by the relative abilities of different macrophage pools to compete for the resident macrophage tissue niche^4^. In addition, previous studies have indicated that the extent to which the functional properties of adult monocyte-derived macrophages either differ from or converge with embryonic-derived macrophages is context-specific^4,6,7^. Under basal conditions, the resident macrophages within a given tissue often exhibit patterns of gene expression which are indistinguishable from one another even when the cells have a different ontogeny^4^. In this regard, adult monocyte-derived macrophages can in some cases be long-lived and take on features more typically associated with homeostatic embryonic origin macrophages^3,5,34^. On the other hand, inherent differences between bone marrow (monocyte)-derived and bone marrow-independent macrophages, possibly based on epigenetic alterations^37^, can become manifest in pathological circumstances. For example, in the mdx mouse model of Duchenne muscular dystrophy, adult bone marrow-derived macrophages are epigenetically modified and appear to play a predominant role in early disease pathogenesis^37–39^.

In conclusion, this study employed diaphragm shielding in chimeric mice to delineate macrophage dynamics in the diaphragm under conditions of basal homeostasis, acute injury, and subsequent recovery. This experimental approach should allow for new insights into the functional characteristics of bone marrow-dependent and bone marrow-independent macrophages in the diaphragm under various conditions. For example, future investigations may discern how the numbers of different macrophage origin subsets are controlled in the tissue, their respective transcriptomic and epigenomic signatures, and the specific roles they fulfill along the spectrum between acute skeletal muscle injury and either successful or unsuccessful repair. The use of diaphragm-shielded chimeras will also be useful for studying chronic pathologies associated with pre-clinical models of human disease such as the mdx (Duchenne muscular dystrophy) mouse, where the diaphragm is often considered the muscle of choice for testing newly emerging immunomodulatory and gene restoration therapies^28^.

## Methods

### Experimental animals

The experimental protocols were approved by the McGill University Health Centre Animal Ethics Committee (protocol number 3480). Experiments were conducted in accordance with regulations set forth by the Canadian Council on Animal Care and the ARRIVE guidelines. C57BL/6J CD45.2 and CD45.1 allele, and CCR2-deficient (CCR2−/−) mice were obtained from The Jackson Laboratories (Bar Harbor, ME, USA). Male mice were housed under specific pathogen-free conditions in a light- and temperature-controlled environment, and were acclimated in our animal facility for 7 days prior to experimentation. Trained personnel provided appropriate care and maintenance, including regular monitoring, to ensure the well-being of the animals throughout the study.

### Bone marrow transplantation

To establish chimeric mice, whole body X-ray irradiation was performed on 6 week old male CD45.1 mice using the X-RAD SmART irradiator (Precision X-ray, USA) either with or without diaphragm shielding with a protective lead bar. Fractionated irradiation was carried out using two 6 Gy doses, administered 4 h apart with parameters set at 225 kV, 13 mA, and 1.0265 Gy/min^40^. Twenty-four hours after the second irradiation session, CD45.1 mice received intravenous injections of bone marrow cells from age- and sex-matched CD45.2 mice (4 × 10^6^ cells for unshielded mice with complete bone marrow ablation and 2 × 10^7^ bone marrow cells for shielded mice with partial radioprotection of the underlying bones) suspended in 200 μl of RPMI (Wisent Cat. #350-000CL, Quebec, Canada). CD45.2 bone marrow cells were collected by flushing the bones with ice-cold RPMI, followed by filtration through a 70 μm cell strainer (ThermoFisher, Cat. #22363458, Ontario, Canada). The cell suspension was then centrifuged at 500*g* for 5 min at 4 °C, and the pellet was resuspended in RPMI at the desired concentrations. To prevent infections, recipient mice were treated with 1% enrofloxacin (50 mg/ml; Bayer, CA, USA) in their drinking water for 7 days after irradiation. Subsequently, the mice were allowed to recover for 8 weeks to allow for complete bone marrow reconstitution.

### Parabiosis

Parabiosis surgeries were performed between CD45.1 wild-type and CD45.2 CCR2−/− strains as we have previously described^41^. To prepare for the surgery, the hair on the side of the mouse to be incised was removed by applying Nair from the neck to the tail. Slow-release buprenorphine (0.1 mg/kg) was administered to the mice before the surgery, and the mice received isoflurane (0.5–2 unit/min) during the surgical procedure. A longitudinal incision was made from the scapula to the femur location. Careful scissor dissection was used to cut the fascia along the incision within a 0.5 cm area. The olecranon of two mice was tied together under the skin using sutures. The incision points, including the starting and ending points, abdominal muscles, and several locations along the incision, were sutured. The remaining part of the incision was closed using staples. The tibia of two mice were tightened by a suture passing through the muscles. Finally, saline (0.5 ml) was injected into each mouse for hydration. The mice were allowed to recover from the surgery and maintained for 5 months prior to sacrifice. Alveolar and interstitial macrophages from the lung served as controls for the expected levels of blood monocyte-independence and dependence, respectively^42^ (see Supplementary Fig. 1).

### Diaphragm injury

Mice were anesthetized using isoflurane for the procedure. To induce acute diaphragm injury, the mice first underwent laparotomy to expose the abdominal surface of the diaphragm as previously described^43^. The fascial layer of the diaphragm was gently abraded, followed by application of a cotton swab immersed in a 10 µM solution of the myonecrotic agent cardiotoxin (Latoxan, Cat. #L8102, Valence, France), which was applied to the entire muscle (30 s for each hemidiaphragm). The diaphragmatic surface was then generously rinsed with saline and the abdominal incision was closed. The animals continued to breathe spontaneously throughout the procedure and recovered well post-operatively.

### Satellite cell isolation and culture

The diaphragm was dissected into small pieces and digested in F12 media (Gibco, Cat. #31966, Ontario, Canada) containing 1% Trypsin (Gibco, 2.5% Trypsin, Cat. #150900468) and 1% Collagenase D (Roche, Cat. # 11088866001, Quebec, Canada) at 37 °C with gentle rotation. After every 1 h of digestion, the supernatant was collected and transferred to a 50 ml Falcon tube on ice, containing 10 ml of FBS (Gibco, Cat. #A5670501). Fresh digestion media was added, and the digestion and collection steps were repeated until all muscle pieces were processed. The collected cell suspensions were filtered through a 70 μm strainer to remove any debris, and then centrifuged at 1800 rpm for 18 min at 4 °C. The resulting cell pellet was resuspended in 80 μl of buffer, which consisted of PBS supplemented with 0.5% FBS. To isolate satellite cells, the resuspended cell suspension was mixed with 20 μl of microbeads from the Satellite Cell Isolation Kit (Miltenyi Biotec, Cat. #130-104-268, CA, USA) and incubated on ice for 15 min. Subsequently, the cell suspension was applied to a rinsed LS column (Miltenyi Biotec, Cat. #130-042-201) placed within a magnetic field (Miltenyi Biotec, Cat. #130-091-051). After applying the suspension, the column was washed twice with 1 ml of buffer to remove unbound cells. The cells passing through the column, which contained the desired satellite cells, were collected by centrifugation at 300 g for 10 min. The resulting cell pellet was then resuspended in 80 μl of buffer and mixed thoroughly with 20 μl of anti-Integrin α7 microbeads (Miltenyi Biotec, Cat. #130-104-261). The suspension was again incubated on ice for 15 min and subsequently transferred to a new rinsed column placed in the magnetic field. To further purify the satellite cells, the column was washed three times with 0.5 ml of buffer, ensuring the removal of any non-specifically bound cells. Finally, the column was removed from the magnetic field, and the satellite cells were collected by washing with 1 ml of full culture media. The full culture media consisted of 20% FBS, 39% DMEM (Gibco, Cat. #31765), 39% F12 (Gibco, Cat. #31966), and 2% Ultrosero G (Cedarlane, Cat. #15950-017, Quebec, Canada), providing the necessary nutrients and growth factors for satellite cell proliferation.

### Satellite cell proliferation and differentiation

To evaluate satellite cell proliferation, the isolated satellite cells were plated onto Matrigel (Corning™, Cat. #354234, NY, USA)-coated 48-well plates in equal numbers for all experimental groups at a density of 10,000 cells per well in full culture media. The culture media was replaced on day 3 post-seeding. On day 3 of culturing, a concentration of 0.03 mg/ml of BrdU (Sigma, Cat. #B5002, MI, USA) was added to the full culture media. At 24 h after BrdU exposure the cells were washed with PBS, then fixed with cold 70% ethanol for 5 min at room temperature, followed by rinsing in 1X PBS. Subsequently, to denature the DNA and expose the incorporated BrdU, the cells were treated with 1.5 M HCl for 30 min and washed again with PBS. To block non-specific binding, the cells were incubated with Protein Block Serum-Free (DAKO, Cat. #X0909, CA, USA) for 60 min. Primary mouse anti-BrdU antibody (Cell Signaling, Cat. #5292S, MA, USA) staining was performed overnight at 4 °C using a 1:1000 dilution in Antibody Diluent (DAKO, Cat. #S3022, CA, USA), followed by rinsing with PBS. The cells were then incubated with rabbit anti-mouse secondary antibody Alexa Flour 488 (ThermoFisher, Cat. #A11059, Ontario, Canada) at a 1:500 dilution in Antibody Diluent for 60 min in the dark. After further PBS rinsing, the cellular DNA was stained with Hoechst (ThermoFisher, Cat. #62249) to visualize nuclei using 1:5000 dilution in PBS for 5 min, followed by additional PBS rinses. The satellite cells were visualized by fluorescence microscopy, and BrdU incorporation was expressed as the percentage of BrdU-positive nuclei divided by total (Hoechst-positive) nuclei^44^. The analysis was performed by a single observer blinded to the identity of the samples.

To induce satellite cell differentiation into myotubes, the cells were switched to media lacking FBS for 4 days. The cells were then fixed by 4% paraformaldehyde (ThermoFisher, Cat. #28908) for 10 min and permeabilized by 0.1 Triton X for 10 min. After blocking, the cells were incubated with F1.652 embryonic myosin antibody (Developmental Studies Hybridoma Bank, IA, USA) at a 1:50 dilution overnight. This was followed by Alex Fluor 488-conjugated anti-mouse secondary antibody 1:500 for 60 min and Hoechst nuclear staining. The differentiated myotubes were visualized by confocal microscopy and individual myotubes were randomly selected for analysis using stereology software ImageJ^45^. The average myotube diameter was determined by measuring the diameter of the myotubes at randomly selected sites. The myotube fusion index was calculated by dividing the number of nuclei within myotubes (containing more than 2 nuclei) by the total number of nuclei as previously described^46^. The analysis was performed by a single observer blinded to the identity of the samples.

### RNA isolation and quantitative real-time PCR (qRT-PCR)

RNA was extracted using TRIzol Reagent (ThermoFisher, Cat# 15596026) according to the manufacturer’s recommendations. RNA purity and integrity were assessed according to the absorbance ratios (A260/280) using a spectrophotometer (BioTek Co., Ltd., Canada). The first strand cDNA was synthesized employing the iScript Supermix (Biorad, Cat #1708840, Canada). Quantitative qRT-PCR was performed in a 10-µL reaction system consisting of 5 µL 2 × SYBR™ Green PCR Master Mix (ThermoFisher, Cat #4368706), 0.5 µL primer mix (10 mM), and 2.5 µL ddH_2_O on a StepOne plus thermal cycler (Applied Biosystems, CA, USA). Briefly, after an initial denaturation step at 95 °C for 10 min, the amplifications were carried out with 40 cycles at a melting temperature of 95 °C for 30 s and an annealing temperature of 60 °C for 1 min, followed by a melting curve analysis at 42 °C. Expression levels of the following genes were determined (see Supplementary Table 1 for PCR primer sequences): MyoD, Myogenin, and the embryonic isoform of Myosin Heavy Chain (MYHC-emb). The relative expression levels of the target genes were determined using the 2^−ΔΔCt^ method^47^. HPRT1 and β-actin were employed as the housekeeping genes.

### Identification of monocytes and macrophages by flow cytometry

Skeletal muscle macrophages and blood monocytes were identified and quantified by flow cytometry as we have previously described in detail^37,38,43^ (see Supplementary Fig. 2). Prior to euthanasia, the mice were anesthetized with isoflurane and blood was drawn by cardiac puncture into a tube containing sodium citrate (Sigma, Cat. #6132-04-3). The heart was then perfused with 20 ml of phosphate-buffered saline (PBS) (Wisent, Cat. #811-425), followed by an additional 20 ml of PBS after severing the dorsal aorta. Red blood cells were lysed using a red blood cell lysis buffer (ThermoFisher, Cat. #A2037300G). The diaphragm, tibialis anterior (TA), and soleus muscles were dissected into small pieces and digested in PBS supplemented with 0.2% collagenase B (Roche, Cat. #11088815001) for 1.5 h to obtain a cell suspension.

Cell populations obtained from the blood and muscles were stained with a viability dye to discriminate live and dead cells. For cell surface markers, the cells were stained at 4 °C in the dark with the following primary antibodies as previously described in detail^37,38,43^: anti-CD45.1 (clone A20, Cat.# 553776), anti-CD45.2 (clone 104, Cat.# 562129), anti-SiglecF (clone E50-2440, Cat.# 562757), anti-CD11c (clone HL3, Cat.# 564986), and anti-CCR2 (Clone 475301, Cat.# 747964) (all from BD Biosciences, NJ, USA); anti-CD11b (clone M1/70, Cat.# 101226), anti-Ly6C (clone HK1.4, Cat.# 128016), anti-F4/80 (clone BM8, Cat.# 123114) and anti-MHC2 (clone M5/114.15.2, Cat#107625) (all from Biolegend, CA, USA). To prevent non-specific binding, the cells were blocked with Fc blocking solution (anti-CD16/CD32, clone Ab93, BD Biosciences, Cat.# 567021). As shown in Supplementary Fig. 2, macrophages in the skeletal muscles were defined as CD45+ (either CD45.1 or CD45.2), SiglecF-negative, CD11c-negative, CD11b+, and F480+. Monocytes in the blood were defined as CD45+ (either CD45.1 or CD45.2), CD11b+, and Ly6C+. The percentage chimerism of bone marrow-dependent macrophages in the muscle was determined by normalizing to the corresponding percentage of blood monocytes in the recipient mouse as previously described^29,30^. For Ki67 staining to assess cellular proliferation, permeabilization buffer (eBioscience, Cat. #00-8333-56, CA, USA) was used before adding the anti-Ki67 antibody (clone SolA15, ThermoFisher, Cat.# 11-5698-82). Cell phenotypes identified by flow cytometry were analyzed using FlowJo version 10.0 software and gating was based on fluorescence minus one (FMO) controls. The analysis was performed by a single observer blinded to the identity of the samples.

### Statistics

Data analysis for significance was performed using Prism GraphPad version 9.0. Distribution of the data was first determined using the Shapiro–Wilk normality test. For normally distributed data, comparisons between two groups were made using Student's unpaired t-test; comparisons between more than two groups were made with one- or two-way ANOVA followed by post-hoc application of the Tukey test to adjust for multiple comparisons. For non-normally distributed data, comparisons between two groups were made using the Mann–Whitney U test; comparisons between more than two groups were made with the Kruskal–Wallis test followed by post-hoc application of Dunn's test to adjust for multiple comparisons. A significance level of p < 0.05 was considered statistically significant. The error bars indicate the standard deviation (SD). All graphs show group mean data with each experimental unit on the graph representing data from a single animal unless specified otherwise. There were no missing or excluded values, and the exact number of animals used for each experiment and all raw data generated or analysed are provided in the individual figures legends and the Supplementary Raw data file, respectively.

### Supplementary Information

Supplementary Information 1.

Supplementary Information 2.
