# Supplementary material for: Dynamic equilibrium of skeletal muscle macrophage ontogeny in the diaphragm during homeostasis, injury, and recovery
Source: Sci Rep. 2024 Apr 21;14:9132. doi: 10.1038/s41598-024-59527-0 (PMC11033281; doi:10.1038/s41598-024-59527-0)
Supplement: Supplementary file 1 — Supplementary Information 1. [file 41598_2024_59527_MOESM1_ESM.docx]

**Dynamic equilibrium of skeletal muscle macrophage ontogeny in the diaphragm during homeostasis, injury, and recovery**

Qian Li ^1^, Feng Liang ^1^, Salyan Bhattarai ^1^, Maziar Divangahi ^1^, Eva Kaufmann ^1 2^,

Basil J Petrof ^1^

^1^Meakins-Christie Laboratories, Translational Research in Respiratory Diseases Program, Research Institute of the McGill University Health Centre, Montreal, Quebec, CANADA

^2^Department of Biomedical and Molecular Sciences, Queen's University，CANADA

*Correspondence to: Basil J. Petrof, MD

Meakins-Christie Laboratories, 1001 Decarie Boulevard, EM3.2224, Montreal, Quebec, Canada, H4A 3J1; Tel: (514) 934-1934, extension 76121; Fax: (514) 933-3962

Email: basil.petrof@mcgill.ca


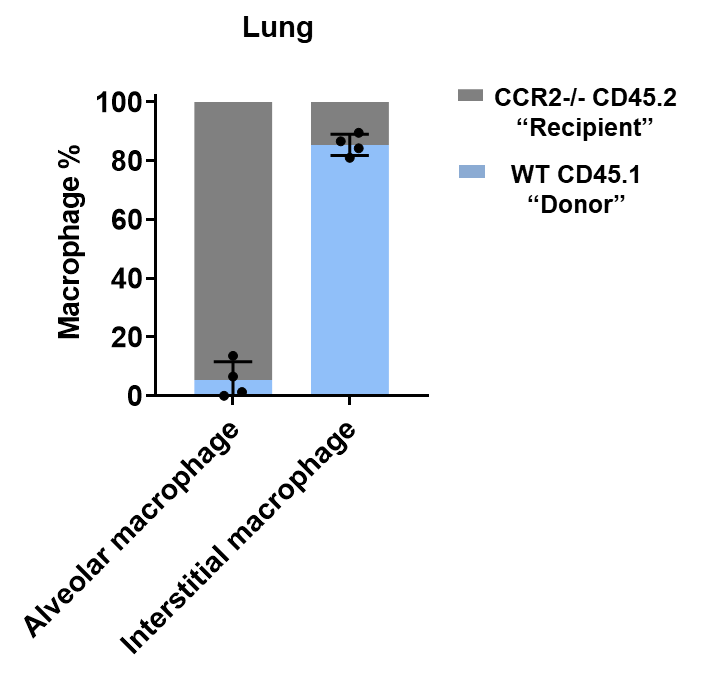


**Supplementary Figure 1. Lung macrophage populations in parabiotic mice**

Alveolar and interstitial macrophages, which are known to have different ontogeny (embryonic progenitors and adult monocytes, respectively), were examined as validating controls in the lungs of CCR2-/- parabiont mice. Alveolar macrophages were defined as Ly6G-, CD11c+, SiglecF+, F480+. Interstitial macrophages were defined as Ly6G-, CD11b+, CD11c-, SiglecF-, F480+.

**
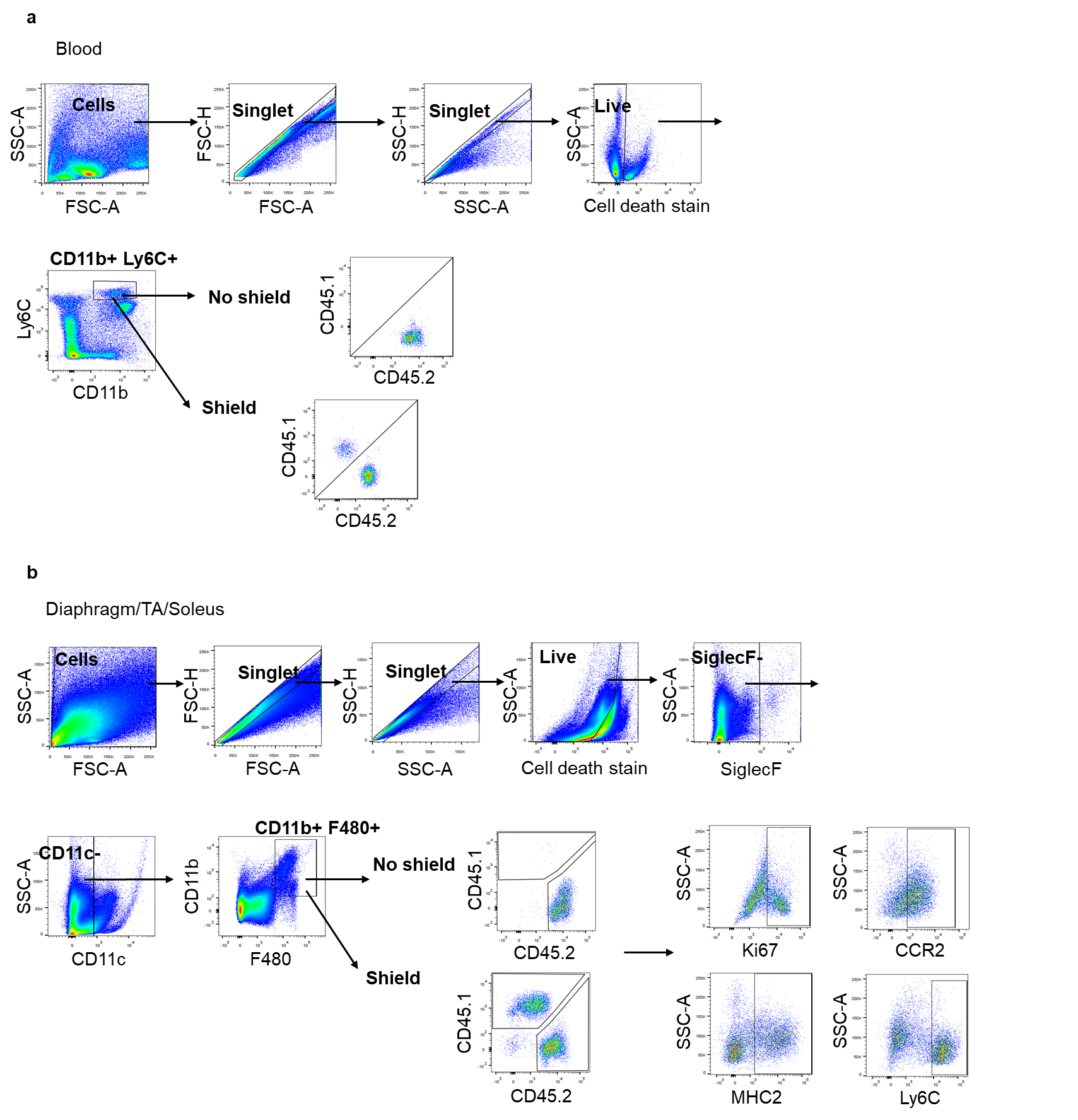
**

**Supplementary Figure 2. Flow cytometry gating strategies for blood and muscles**

1. Blood samples. Two rounds of singlet selection were performed. Live cells were filtered based on CD11b+ and Ly6C+, classifying them as monocytes. The relative proportion of CD45.1+ and CD45.2+ monocytes thus identified was then determined.
2. Muscle samples. Gating was applied after singlet selection and viability assessment, following a similar procedure as with blood samples. Macrophages were identified as both CD11b+ and F480+, while excluding cells which were SiglecF+ (eosinophils) or CD11c+ (dendritic cells). The relative proportion of CD45.1+ and CD45.2+ macrophages thus identified was then determined. Additionally, the expression of Ki67 was examined to assess macrophage proliferation. CCR2, Ly6C and MHC2 and Ly6C were examined to further characterize macrophage phenotype.

| **Gene name** | **F/R** | **Sequence** |
| --- | --- | --- |
| HPRT1 | F | CGCAGTCCCAGCGTCGTGAT |
|  | R | CGAGCAAGTCTTTCAGTCCTGTCCA |
| ACTB | F | CGACAACGGCTCCGGCATGT |
|  | R | TCTGGGCCTCGTCACCCACA |
| MYOD | F | AGAATGGCTACGACACCGCC |
|  | R | GCTGTCTGTGGAGATGCGCT |
| MYOG | F | GAGGAGCGCGATCTCCGCTA |
|  | R | GTCAGCCGCGAGCAAATGAT |
| MYHC-emb | F | GCTCACATATCAGAGTGAGGAGGCA |
|  | R | TCCTCAGCCTGCCTCTTGTAGGA |

**Supplementary Table 1. Primer Sequences for qPCR**

The primer sequences used for gene amplification by quantitative polymerase chain reaction (qPCR) are shown. Forward (F) and reverse (R) primer sequences are listed for each gene.
